# Do patients’ pre-treatment expectations about acupuncture effectiveness predict treatment outcome in patients with chronic low back pain? A secondary analysis of data from a randomised controlled clinical trial

**DOI:** 10.1371/journal.pone.0268646

**Published:** 2022-05-20

**Authors:** Anja Zieger, Alexandra Kern, Jürgen Barth, Claudia M. Witt

**Affiliations:** 1 Institute for Complementary and Integrative Medicine, University Hospital Zurich and University of Zurich, Zurich, Switzerland; 2 Institute for Social Medicine, Epidemiology and Health Economics, Charité–Universitätsmedizin Berlin, Freie Universität Berlin and Humboldt-Universität zu Berlin, Berlin, Germany; 3 Center for Integrative Medicine, University of Maryland School of Medicine, Baltimore, MD, United States of America; Monash University, AUSTRALIA

## Abstract

**Objective:**

This secondary analysis of a randomised controlled patient-blinded trial comparing effectiveness and side effect briefings in patients with chronic low back pain (CLBP) investigated the association between patients’ pre-treatment expectations about minimal acupuncture treatment and pain intensity as outcome during and after the end of the treatment.

**Methods:**

Chronic low back pain patients with a pain intensity of at least 4 on a numeric rating scale from 0 to 10 received eight sessions of minimal acupuncture treatment over 4 weeks. The primary outcome was change in pain intensity rated on a Numerical Rating Scale (NRS 0–10) from inclusion visit to treatment session 4 and to the end of the treatment. Patients’ expectations about the effectiveness of acupuncture were assessed using the Expectation for Treatment Scale (ETS) before randomization. Linear regression was applied to investigate whether patients’ pre-treatment expectations predicted changes in pain intensity during and after treatment.

**Results:**

A total of 142 CLBP patients (40.1 ± 12.5 years; 65.5% female) were included in our analysis. Patients’ pre-treatment expectations about acupuncture treatment were associated with changes in pain intensity after four sessions of minimal acupuncture treatment (*b* = -0.264, p = 0.002), but not after the end of the treatment. This association was found in females and males.

**Conclusions:**

Our results imply that higher pre-treatment expectations only lead to larger reductions in pain intensity in the initial phase of a treatment, with a similar magnitude for both females and males. As the treatment progresses in the second half of the treatment, adapted expectations or other non-specific effects might play a more important role in predicting treatment outcome.

## Introduction

Patient expectations in the context of medical treatment have received increasing attention in research in recent years, with a growing body of literature suggesting their influence on clinical outcomes. Associations of patient expectations and treatment outcomes have been shown for a variety of medical conditions including heart disease [1], post-chemotherapy nausea in cancer [2], back pain [3–5] and neck pain [6], as well for different therapeutic interventions including surgery [7–11], psychotherapy [12], chiropractic care [5], physical therapy [6], massage [13] and acupuncture [13,14]. However, relatively few studies have investigated the predictive value of patient expectations on outcomes of acupuncture treatment, with inconsistent results [15].

Acupuncture is part of Chinese Medicine, and data from a large patient data meta-analysis suggests that acupuncture is statistically significantly superior to sham acupuncture and no acupuncture control conditions. The differences between real acupuncture and sham acupuncture, however, were not clinically meaningful [16]. The authors concluded that factors other than the acupuncture point-specific effect notably contribute to the effectiveness of acupuncture in reducing pain [16–18]. Data from other chronic pain conditions showed that 47–91% of the measured pain reduction from interventions might be attributable to contextual factors, with 85% for acupuncture [19].

Those non-specific contextual factors are complex sets of internal, external or relational elements, including patient’s expectations, history, baseline characteristics, type of treatment, treatment setting, and clinician’s behaviour [20,21]. Contextual factors can trigger specific treatment expectations [22,23] which have been identified as one of the major components contributing to a placebo response [24]. Patient treatment expectations could therefore contribute to the analgesic effects of acupuncture.

Previous studies investigating the effect of expectations on outcomes of acupuncture were heterogeneous in terms of study design, pain condition, outcome measures, and measurement of expectation, which makes it difficult to draw definitive conclusions. Some studies found strong effects [14,25]; others, however, found no effects [26–28], or mixed effects [13,29]. Due to the heterogeneity of the studies, the authors of two systematic reviews did not pool studies in a meta-analyses to estimate the magnitude of the effect of patient expectations on outcomes [15,30]. From the available studies, five included patients with low back pain [13,14,26,27,29] which corresponds to the sample of our secondary analysis. The study by Kaloukalani et al. [13] found that patients who had higher pre-treatment expectations that their acupuncture treatment would be helpful were more likely to have clinically important improvements in function after 10 weeks. The authors did not find an association between general expectations and treatment outcome. The exploratory analysis by Thomas et al. [27] including 241 patients did not support that positive beliefs regarding acupuncture were associated with better treatment outcomes after 2 years. Sherman and colleagues [26] did not find patients’ general expectations or specific expectations for acupuncture to be predictive of functioning after 8 or 52 weeks in acupuncture-naïve patients. Myers et al. [29] investigated recovery expectations in patients with acute low back pain and found that patients’ general expectations for recovery but not the specific treatment expectations were associated with greater functional improvements from baseline to 5 and 12 weeks. The pooled analysis by Linde et al. [14] combined data from four large trials and included 864 chronic pain patients (219 patients with low back pain) who received either acupuncture or sham acupuncture treatment [14]. The analysis showed a significant influence of patients’ pre-treatment expectations on Pain Disability Index as outcome across all four chronic pain conditions. The influence of patient expectations on treatment outcome in the minimal acupuncture group, however, was less consistent compared to the real acupuncture group. The inconsistent results might be due to the wide range of items and response scales used to measure patient treatment expectations in acupuncture trials, which underline the need of a more standardised assessment approach, using standardised questions and response scales [15], and using a clear definition and a sharp distinction of patient expectations and associated terms [31].

To expand our understanding of the association between patients’ pre-treatment expectations and treatment outcome, we performed this secondary data analysis based on the data from a randomised controlled clinical trial [32]. The trial aimed to investigate whether patients’ treatment expectations and reported adverse side effects could be affected by different briefing contents in a 2 x 2 factorial design (effectiveness briefing x side effect briefing) and to assess the influence of the manipulated expectations on treatment outcomes in patients with CLBP. All patients received the same standardised minimal acupuncture treatment consisting of eight treatment sessions within 4 weeks. The briefing intervention (two oral briefing sessions and written materials) was standardised and delivered before the acupuncture treatment and boostered through additional booster emails during the acupuncture treatment. No difference was found in patients’ expectations regarding the effectiveness of the acupuncture treatment between the intervention groups (-0.16; 95% CI -0.81 to 0.50, p = 0.64) after receiving the briefing intervention. There was also no evidence for a difference in pain intensity at the end of the acupuncture treatment. For this analysis, only patients’ pre-treatment expectations before seeing our study physician were of interest.

With this analysis, we wanted to answer the questions whether patients’ pre-treatment expectations about acupuncture treatment predict changes in pain intensity (a) after four treatment sessions and (b) after the end of the treatment. We hypothesised that patients with higher expectations about the effectiveness of acupuncture treatment were more likely to experience reductions in pain intensity than patients with lower expectations at both time points independently of sex.

## Methods

### Study design

This secondary analysis is based on the data from a randomised controlled patient-blinded trial comparing a regular expectation briefing with a high expectation briefing (factor effectiveness) and a regular side effect briefing with an intense side effect briefing (factor side effects) using a 2 x 2 factorial design in patients with chronic low back pain (CLBP).

All patients received eight sessions of minimal acupuncture treatment within 4 weeks. The standardised briefing intervention with the study physician took place before the first acupuncture session (lasting approximately 30 minutes) and before the third acupuncture session (lasting approximately 15 minutes). Two booster emails were sent after the third and sixth acupuncture session. Because there were no significant differences between the two expectation briefing groups in expectation and pain outcomes, all patients were pooled for this analysis [32]. The sample size was calculated for the primary trial and the sample size calculation showed that the study needed 128 patients to have 80% power (2-sided test; α = 0.05) to show a clinically meaningful effect with an SMD of 0.5 on the patients’ treatment expectations. Considering dropouts, a total of 150 patients were planned.

The trial was conducted in accordance with the Declaration of Helsinki [33] and the International Conference on the Harmonisation of Good Clinical Practice. It was approved by the Cantonal Ethics Committee of Zurich (BASEC Nr. 2015–0511) and registered with the German Clinical Trials Register https://www.drks.de (DRKS00010191).

### Study population

Patients were recruited via email newsletters directed to the staff of the University Hospital Zurich and other university institutions; by posters and flyers in university buildings, fitness gyms and practises of physicians; and via the institutes´ website. First patient was recruited May 5, 2016, and last patient completed the study December 6, 2017. Individuals interested in study participation received information about the study and underwent a preliminary telephone screening regarding location, duration, and intensity of their back pain.

**Fig 1:**
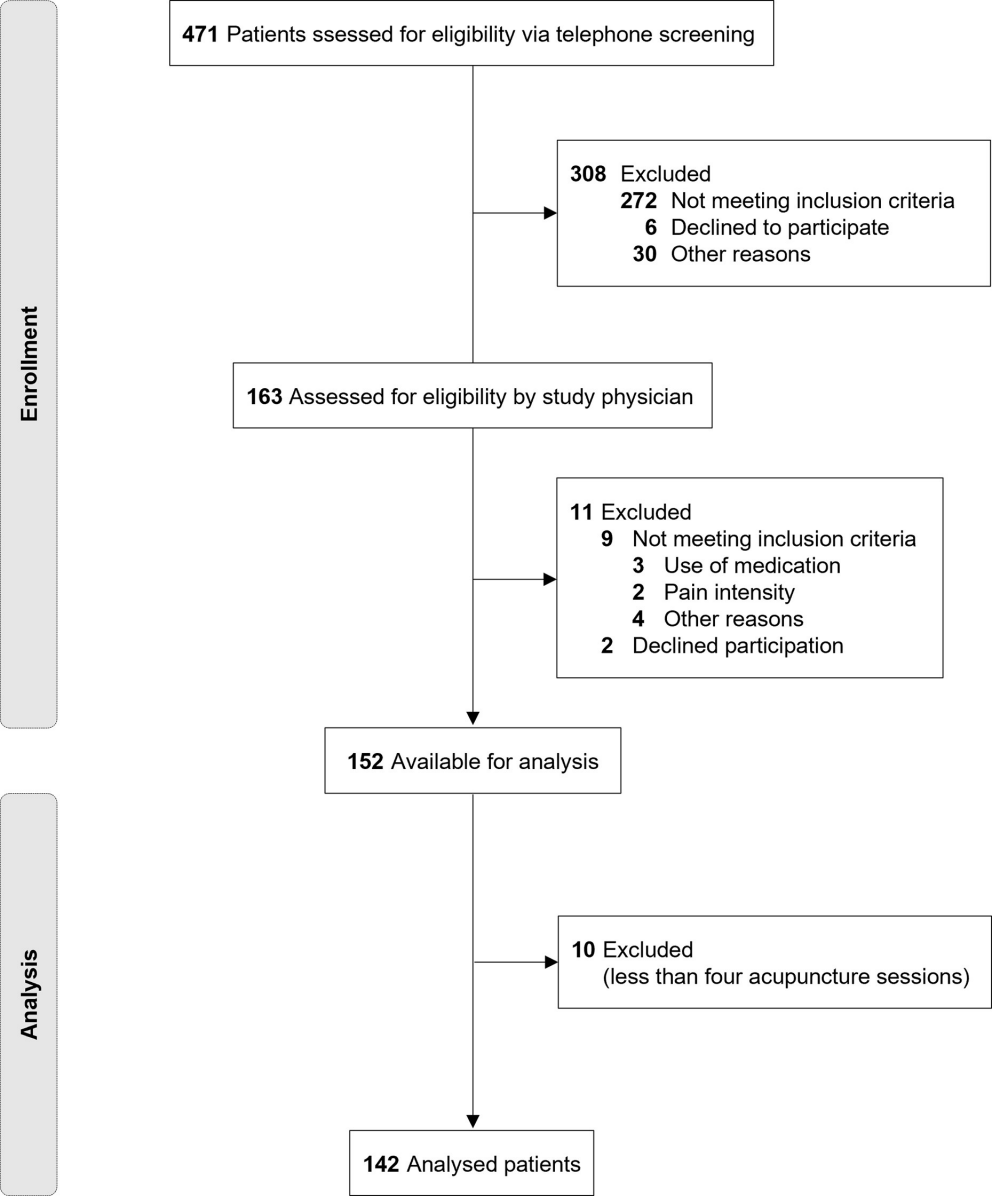
Flow chart of patient inclusion.

We included male and female patients between 18 and 65 years of age with clinically diagnosed CLBP [34] with an average pain intensity of ≥ 4 (NRS 0–10) in the last 7 days and with pain on at least 4 out of 7 days per week within the last six months. Patients were excluded if they had an acupuncture treatment within the last 12 months (see Fig 1).

All patients gave written informed consent before they were randomised into the trial. All randomised patients who received at least four sessions of acupuncture treatment were included in the analysis.

### Treatment

All patients received the same standardised minimal acupuncture treatment, which had been used successfully before in a trial and was shown to be more effective than no treatment [35]. The treatment consisted of eight acupuncture sessions of 45 minutes duration applied over a period of 4 weeks, preferably with two sessions per week. Fine needles (30 mm in length) were superficially placed at six predefined points bilaterally that were not traditional acupuncture points and manually stimulated at the beginning and end of the treatment session, while the sensation of de qi was avoided. Needles were placed in the forearm and remained there for three minutes before they were placed in the remaining points where they stayed for 25 minutes. The non-acupuncture points were appointed in a consensus procedure for previous trials and resulted in a reduction in pain severity of 50% in 38% of the patients with chronic low back pain [35,36]. Treatment was delivered by three therapists who were specifically trained to deliver this treatment in a very standardised manner and supervised by an MD acupuncturist. More details about the intervention are provided in our earlier publication [32].

Patients were allowed to treat chronic low back pain with non-steroidal anti-inflammatory drugs if required. The use of pain medication had to be documented in a medication diary. All patients were informed that they would receive a treatment that was shown to be beneficial for chronic low back pain.

## Measures

### Patient expectations

Patients’ expectations about acupuncture treatment effectiveness were assessed using the Expectation for Treatment Scale (ETS) [37] before randomization. In this study, patient expectations were defined as the treatment-related outcome expectations and as the beliefs that a treatment would have a positive or negative effect on health status [38].

The ETS is a well-validated scale consisting of 5 items, each rated on a 4-point scale ranging from 1 (partially disagree) to 4 (definitely agree). The total score ranging from 5 to 20 is the sum of all responses, and higher values indicate higher expectations about acupuncture treatment. The scale showed acceptable internal consistency (α = 0.77) in our patient population, and the ETS score was normally distributed.

### Pain intensity

Pain intensity during the previous 7 days was assessed at the inclusion visit, after acupuncture session 4, and after the end of the treatment using an 11-point NRS ranging from 0 (no pain) to 10 (worst imaginable pain) [39–41]. Our primary outcome was change in pain intensity from inclusion visit to treatment session 4 and to the end of the treatment, calculated by subtracting the NRS score of the inclusion visit from the NRS score after session 4 and after the end of the treatment, respectively. A negative change score indicated a beneficial effect of the treatment over time.

### Further measures

Data on patient demographics, including age, sex, education, and employment status, were obtained via the baseline questionnaire.

Optimism and pessimism were assessed using the German version of the Life Orientation Test—Revised (LOT-R) [42]. The 10-item questionnaire consists of three positively formulated items, three negatively formulated items and four filler items. Each item was rated on a 5-point Likert scale ranging from 0 (strongly disagree) to 4 (strongly agree). As recommended by Glaesmer and colleagues, optimism and pessimism scores were generated [42]. The optimism subscale was calculated by summing the three positively formulated items; the pessimism subscale score was calculated by summing the three negatively formulated items. Both subscales have scores ranging from 0 to 12, with higher scores indicating more optimism or more pessimism. The Cronbach´s alpha in our patient population was acceptable for optimism (α = 0.74) and questionable for pessimism (α = 0.69).

Self-reported health was assessed at baseline using the 29-item short-form of the Patient-Reported Outcomes Measurement Information System® (PROMIS; www.nihpromis.org). The PROMIS Profile-29 combines the 4-item short forms from the seven PROMIS domains (depression, anxiety, physical function, pain interference, fatigue, sleep disturbance, and ability to participate in social roles and activities) and a single item on pain intensity [43]. The items of each domain are rated on a 5-point Likert scale. The total score of each domain was converted to a standardised T-score, with greater severity indicated by higher scores for negatively worded concepts (anxiety, depression, pain interference, fatigue, sleep disturbance) and lower scores for positively worded concepts (physical function and ability to participate in social roles and activities).

Information about previous acupuncture treatment and the success of any previous treatment was obtained during the telephone screening.

Pain bothersomeness during the previous 7 days was assessed at baseline, after acupuncture session 4 and after the end of the treatment using an 11-point NRS ranging from 0 (no pain) to 10 (worst imaginable pain). The duration of pain was assessed at the inclusion visit by asking patients about the onset (month and year) of chronic low back pain and calculated by subtracting the date of the onset of pain from the date of the inclusion visit.

### Statistical analysis

The analysis followed a prespecified analysis plan which was finalised and signed prior to starting the analysis. Descriptive statistics, including frequencies and percentages (%) for categorical variables and mean and standard deviation (SD) for continuous variables, were used for patient characteristics and measures of interest. To assess the differences between females and males, Chi-square test was used for categorical variables and one-way ANOVA was used for continuous variables. No adjustments for multiple comparisons were done.

An independent t-test was used to compare patient pre-treatment expectations between patients who previously received acupuncture treatment and those who did not have acupuncture treatment before. A dependent t-test for paired samples was used to investigate the differences between pain intensity at the inclusion visit, after four sessions of acupuncture, and after completion of the treatment. Cohen´s d for repeated measurements was calculated to measure the effect size between pre- and post-treatment scores [44].

Pearson correlations were used to test for the associations of the sociodemographic (sex and age), previous experience (previous acupuncture treatment, number of sessions of last treatment, success of last treatment), pain (pain intensity, pain bothersomeness, duration of pain), self-reported health (PROMIS), personality traits (LOT-R), and briefing group variables with the primary outcome change in pain intensity and patient expectations.

Unadjusted linear regression was used to estimate standardised beta coefficients (*b*) to determine whether expectations were associated with a change in pain intensity after four acupuncture sessions or after the end of the treatment. Because our primary focus was the unadjusted analysis, adjusted regression models were only run if any of the variables included in the Pearson correlations were found to have an association with change in pain intensity with a p-value of ≤ 0.10. Analyses were performed for the total sample and stratified by sex.

Missing variables used in the linear regression analyses were imputed using multivariate imputation by chained equations using the R package MICE [45] with the percentage of missing values for the total sample ranging from 0.7% to 2.1%. Sensitivity analyses were conducted without any missing data imputation procedure. All analyses were performed using the statistical software R version 3.3.2 [46].

## Results

Of the 152 patients who were randomised into the trial, 142 patients had at least four acupuncture sessions and were included in this analysis (see Fig 1). The mean number of acupuncture sessions received by those patients was 7.9 (± 0.6).

As displayed in Table 1, our patient population tended to be well-educated, and two-thirds of our patients were female. The mean CLBP pain duration was 8.3 (± 8.7) years, and the mean pain intensity score in the past 7 days was 5.4 (± 1.4) and represented moderate levels of pain.

**Table 1:** Patients’ demographic and clinical characteristics stratified by sex.

	n*	Total (n = 142)	Female (n = 93)	Male (n = 49)	p-value
**Age (years)** ^1^ **, *mean* (SD)**	142	40.1 (12.5)	40.6 (13.1)	39.2 (11.2)	0.533
**Education**^2^ **(%)**					0.27
School ≤ 10 years	3	2.1	1.1	4.1	
School ≥ 12 years	80	56.3	60.2	49.0	
University	59	41.5	38.7	46.9	
**Employment status**^2^ **(%)**					0.217
Employed	110	77.5	81.7	69.4	
Unemployed	3	2.1	1.1	4.1	
Student	22	15.5	11.8	22.4	
Other	7	4.9	5.4	4.1	
**First language**^2^ **(%)**					0.918
German	118	83.1	83.9	81.6	
Other	24	16.9	16.1	18.4	
**Previous experience**					
Previous acupuncture treatment^2^ (%)	65	45.8	51.6	34.7	0.107
Success of last acupuncture treatment^1^^,^^b^, *mean* (SD)	58	5.7 (3.1)	5.9 (3.1)	5.1 (3.1)	0.409
**Pain** ^1^					
Pain intensity^a^, *mean* (SD)	142	5.4 (1.4)	5.4 (1.4)	5.3 (1.2)	0.547
Pain bothersomeness^a^, *mean* (SD)	142	5.5 (1.9)	5.3 (1.9)	5.8 (1.9)	0.206
Duration of pain (months), *mean* (SD)	142	100.0 (104.7)	107.8 (110.4)	85.3 (92.4)	0.227
**ETS Expectation** ^1^ ^,^ ^c^ **, *mean* (SD)**	140	12.2 (3.3)	12.3 (3.3)	12.0 (3.1)	0.620
**Self-reported health** ^1^					
PROMIS Anxiety, *mean (SD)*	141	54.4 (8.0)	53.9 (7.5)	55.4 (8.8)	0.316
PROMIS Depression, *mean (SD)*	142	52.1 (8.0)	52.4 (7.9)	51.6 (8.4)	0.590
PROMIS Ability to participate in social roles, *mean (SD)*	139	49.0 (7.5)	49.4 (7.2)	48.2 (8.1)	0.351
PROMIS Fatigue, *mean (SD)*	142	54.1 (9.1)	54.5 (9.2)	53.4 (8.8)	0.501
PROMIS Pain interference, *mean (SD)*	141	58.3 (4.8)	58.4 (4.9)	58.2 (4.6)	0.848
PROMIS Physical function, *mean (SD)*	142	46.1 (5.6)	46.0 (5.6)	46.4 (5.6)	0.725
PROMIS Sleep disturbance, *mean (SD)*	141	51.2 (8.7)	51.2 (8.6)	51.2 (8.9)	0.982
**Personality traits** ^1^ ^,^ ^d^					
LOT-R Optimism, *mean (SD)*	140	8.8 (2.4)	8.9 (2.4)	8.5 (2.5)	0.355
LOT-R Pessimism, *mean (SD)*	139	3.4 (2.3)	3.1 (2.2)	3.9 (2.2)	0.045

Abbreviations: ETS, Expectation for Treatment Scale; LOT-R, Life Orientation Test—Revised; NRS, numeric rating scale; PROMIS, Patient-Reported Outcomes Measurement Information System®.

*n depicts the number of (available) measures.

^1^ One-way ANOVA used for continuous variables.

^2^ Chi-square test used for categorical variables.

^a^ Measured on a numerical rating scale (range 0–10; higher scores indicate more pain or bothersomeness).

^b^ Measured on a numerical rating scale (range 0–10; higher scores indicate greater success).

^c^ ETS is scored from 5 to 20; higher scores indicate greater expectations for success.

^d^ Measured with the Life Orientation Test-Revised (range 0–12; higher scores indicate greater optimism or pessimism).

Patients in our study had rather low pre-treatment expectations with a mean ETS score of 12.2 (± 3.3). Patient pre-treatment expectations were significantly higher in patients who had a previous acupuncture treatment compared to patients who did not have acupuncture treatment before (13.1 ± 3.7 (n = 65) versus 11.5 ± 3.5 (n = 77), p = 0.003). Additional factors associated with higher patient pre-treatment expectations were pain intensity (r = 0.34, SE = 0.08, p < 0.001), pain bothersomeness (r = 0.23, SE = 0.083, p = 0.007) and fatigue (r = 0.2, SE = 0.084, p = 0.02).

**Fig 2:**
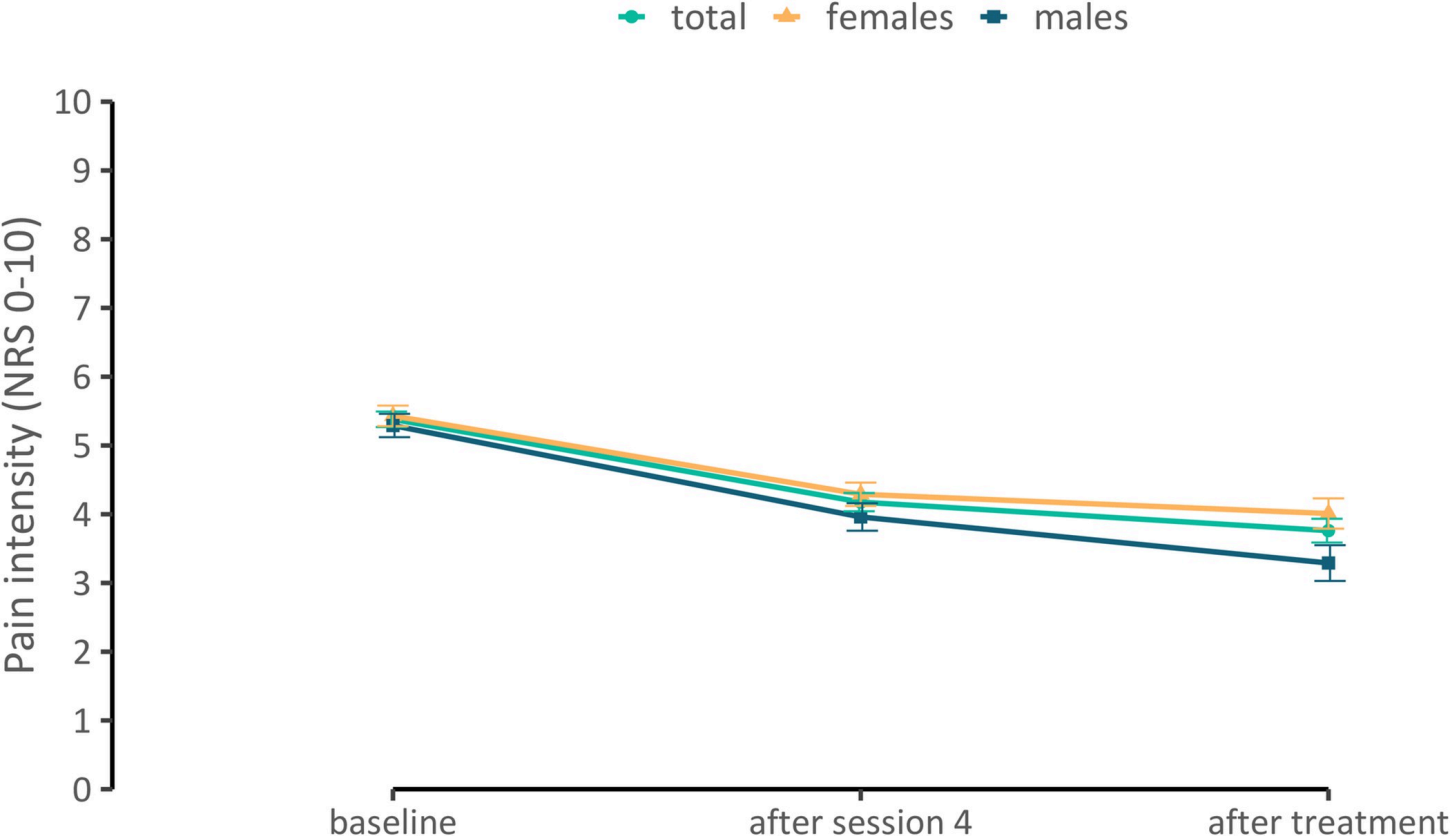
Pain intensity at baseline, after four sessions and after the end of the acupuncture treatment displayed for total sample, males, and females.

The mean pain intensity (NRS 0–10) significantly decreased by 1.20 points (95% CI 0.952–1.457, p < 0.001) after four acupuncture sessions and by 1.62 points (95% CI 1.304–1.936, p < 0.001) after the end of treatment, resulting in a pre-post Cohen’s d of 0.72 and 0.71, respectively (see Fig 2).

As displayed in Table 2, patients’ expectations were significantly associated with improvements in pain intensity after four acupuncture sessions. For each 1-point increase in patients’ expectations on the ETS, there was a 0.264-point improvement in pain (p = 0.002). In other words, patients who had a 4-point higher score on the ETS had a 1-point improvement in pain intensity on the 0–10 NRS. This association was equally found for females (*b* = -0.258, SE = 0.101, p = 0.013) and males (*b* = -0.299, SE = 0.14, p = 0.039). After adjusting for baseline pain bothersomeness and anxiety, the standardised estimate of beta of the total sample decreased to -0.219 (SE = 0.083), meaning that for each 1-point increase in patients’ expectations, there was a 0.219-point improvement in pain. This value, however, retained statistical significance (p = 0.01). This significant association was also found in females (*b* = -0.227, SE = 0.106, p = 0.035) but the association was not significant in males. Even after adding the remaining variables to our analysis (expectation briefing group, side effect briefing group, PROMIS subscales, LOT-R, PSM and side effect score) patient expectation still had a significant impact on change in pain intensity for the total sample (*b* = -0.257, SE = 0.039, p = 0.005). No significant associations were found between patients’ expectations and improvement in pain intensity between inclusion visit and the end of the treatment at the end of week four.

**Table 2:** Results of linear regression analysis for change in pain intensity as primary outcome and patient expectation as predictor presented as standardised betas.

	Change in pain intensity after treatment session 4					Change in pain intensity after treatment				
	*b*	SE	p-value	R^2^	n	*b*	SE	p-value	R^2^	n
**Expectation at baseline (unadjusted)**										
Total	-0.265	0.082	**0.001**	0.07	142	-0.145	0.084	0.087	0.021	142
Females	-0.258	0.101	**0.013**	0.066	93	-0.138	0.104	0.188	0.019	93
Males	-0.299	0.14	**0.039**	0.089	49	-0.169	0.147	0.257	0.028	49
**Expectation at baseline (adjusted)** *										
Total	-0.219	0.083	**0.009**	0.105	142					
Females	-0.227	0.106	**0.035**	0.083	93					
Males	-0.232	0.147	0.123	0.164	49					

Significant results are marked in bold. Negative sign indicates an impact of expectations on an improvement in pain intensity.

*Analysis was adjusted for pain bothersomeness and anxiety.

## Discussion

This analysis shows that in patients with chronic low back pain, patients’ pre-treatment expectations about acupuncture were predictive of improvements in pain intensity after four treatment sessions but not after the end of the treatment. This effect was found in females and males. Pain intensity decreased over the course of treatment. The observed pre-post pain intensity reduction was approximately 30%, which is usually regarded as clinically meaningful [47–50].

The inconsistent findings of the previous studies analysing the relationship between patient expectations and acupuncture treatment outcomes in patients with low back pain might be due to differences in how expectations and outcomes were defined and measured and how patients were recruited. Laferton et al. [51] pointed out that this heterogeneity in measurements makes conclusions difficult. Most studies measured general expectations for improvement and treatment-specific expectations using a single item each [13,26,27,29]. Timepoints for assessing expected general improvement ranged from 6 weeks [29] up to one year [26]. Treatment-specific expectations assessed how helpful patients believed the treatment would be for their current back pain [13,26,29], if patients believed that acupuncture might be helpful [27] or what the patients personally expected from the treatment [14]. Responses were mostly dichotomised [13,14] or split into three groups [26]. Although it is not known how dichotomization affects the predictive strength of expectation [15], dichotomization always results in a loss of information [52,53]. To account for the need for a more standardised approach to measure expectations [15,27,30], we used the ETS as validated scale to measure pre-treatment expectations of acupuncture treatment, and we included the original scale in our analysis.

Only two studies used pain intensity as an outcome, which was measured after 8 weeks [14] or 24 months [27]; the remaining studies used function as an outcome, which was measured after 5 and 12 weeks [29], after 8 and 52 weeks [26] or after 10 weeks [13]. None of the studies investigated the effect of patient expectations on short-term improvements, e.g., after 2 weeks of treatment, as our study did. The two studies using pain intensity as an outcome used different assessment tools: the SF-36 Bodily Pain Scale [27] or a VAS 100 mm [14]. We used an NRS (0–10), which is a valid measure and is highly correlated with the VAS [40,54]. Following the approach of Myers et al. [29], we used a change score as the dependent variable. While Myers found only an association between general expectations and treatment outcome, we found an association between treatment-specific expectations and treatment outcome after four treatment sessions. This might be explained by the difference in patient populations, i.e., acute vs. chronic low back pain. While patients with acute pain expect their pain to improve independently of the type of treatment, patients with chronic pain might have unsuccessfully tried different treatment options and therefore have specific expectations for acupuncture that predict improvements in pain in the first half of the treatment.

Of note were the relatively low baseline expectations about the effectiveness of acupuncture to treat chronic low back pain in our patient population, which can potentially be explained by the fact that most of our patients were recruited through flyers and not referred from their general practitioner. They may not have chosen acupuncture specifically because of their expectations about this treatment but rather because they coincidentally became aware of the possibility to try it. The 46% of the patients who had a previous acupuncture treatment had significantly higher expectations than the acupuncture-naïve patients. Although previous acupuncture treatment is an important predictor of further acupuncture usage and might therefore yield higher expectations [55], the higher expectations cannot be solely explained by patients’ previous experience with acupuncture as suggested by Sherman et al. [26]. Patients in the pooled analysis by Linde et al. [14] seemed to have higher expectations than patients in our trial, but only 30% (vs. 46% in our trial) had previous experience with acupuncture. Additional contextual factors [20] might therefore have a larger influence on patient pre-treatment expectations than has previously been assumed.

Patient expectations predicted the clinically non-meaningful improvements in pain intensity after four sessions but not the clinically meaningful improvements in pain intensity after eight sessions, the end of the acupuncture treatment. Thus, patients with higher expectations improved more compared to patients with lower expectations in the first half of the treatment; however, the overall improvements from the treatment were independent of patients’ expectations. Although not clinically meaningful, most improvements in pain occurred during the first half of the treatment. The additional improvements in the second half of the treatment were comparatively small. This is in line with results presented by Pach et al. [56], who found the largest improvements in the initial part of treatment for CLBP patients receiving standardised and individualised acupuncture treatment. While expectations seem to play an important role in the initial treatment phase, other factors such as the physiological effects of minimal acupuncture (non-acupuncture point specific effects), non-specific effects such as natural healing, or response bias might be relevant for explaining the additional improvements in pain intensity after the end of the treatment. In addition, expectations can be modified through direct experience [57]. Experiences in the first half of the treatment might lead to adapted expectations during subsequent treatment, which in turn might better predict additional improvement in the second half of the treatment.

Patient pre-treatment expectations can be regarded as meaningful in the initial part of the treatment, but factors other than expectations might be more relevant in predicting long-term improvements in pain intensity. The assessment of patient expectations could be valuable for clinicians who wish to predict their patients’ prognoses for a specific treatment since both sides might have different views on expected benefits [58]. Future work should not only focus on treatment outcome after the treatment but also investigate the predictive value of patients’ expectations during treatment. In addition, future clinical trials should be encouraged to assess pre-treatment expectations with a validated measurement tool such as TEX-Q [51] or ETS [37] and to report the results irrespectively of findings to improve our understanding of patient expectations and its predictive value on clinical outcomes.

Our analysis had several strengths: It used a validated scale to measure patient expectations with good internal validity; it used data from a trial with a highly standardised setting, high adherence to treatment, and high follow-up rates; it used common pain outcomes; and it had approximately equal proportions of patients with and without previous acupuncture experience. One potential limitation of the study is that patients were mostly recruited via posters and flyers, and these patients might differ from patients seeking treatment by an acupuncturist in a usual care setting. Also, we did not assess the interventions used by patients as non-pharmacological usual care intervention. They might, however, also have an impact on pain intensity during and after the treatment. Eventually, the study was a single-centre study conducted in the metropolitan area of Zurich, and the sample was well-educated. Our patients might therefore not be representative of the general back pain population of Switzerland.

## Conclusion

Findings from this analysis add further insight into the association between patients´ pre-treatment expectations and pain intensity as outcome in patients with chronic low back pain. We have not been able to show that pre-treatment expectations enhance treatment outcomes after the end of the treatment. Our results imply that higher pre-treatment expectations only lead to greater reductions in pain intensity in the initial phase of a treatment with a similar magnitude for both females and males. As the treatment progresses, adapted expectations or other non-specific effects might play a more important role in predicting treatment outcome.

## Supporting information

S1 Fig Treatment flow. (TIF)

S1 Table Pearson correlations to test for the associations of all relevant variables and change in pain intensity as primary outcome. (PDF)

S2 Table Sensitivity analysis of linear regression for change in pain intensity as primary outcomes and patient expectation as predictor additionally adjusted for expectation and side effect briefing group. (PDF)

S3 Table Sensitivity analysis of linear regression for change in pain intensity as primary outcomes and patient expectation as predictor additionally adjusted for expectation and side effect briefing group, PROMIS, LOT, PSM and side effects. Significant results are marked in bold. Negative sign indicates an impact of expectations on an improvement in pain intensity. *Analysis was adjusted for pain bothersomeness, anxiety, expectation briefing group, side effect briefing group, PROMIS, LOT, PSM and side effect score.
(PDF)
