# Supplementary figures and images for: Do patients’ pre-treatment expectations about acupuncture effectiveness predict treatment outcome in patients with chronic low back pain? A secondary analysis of data from a randomised controlled clinical trial
Source: PLoS One. 2022 May 20;17(5):e0268646. doi: 10.1371/journal.pone.0268646 (PMC9122231; doi:10.1371/journal.pone.0268646)

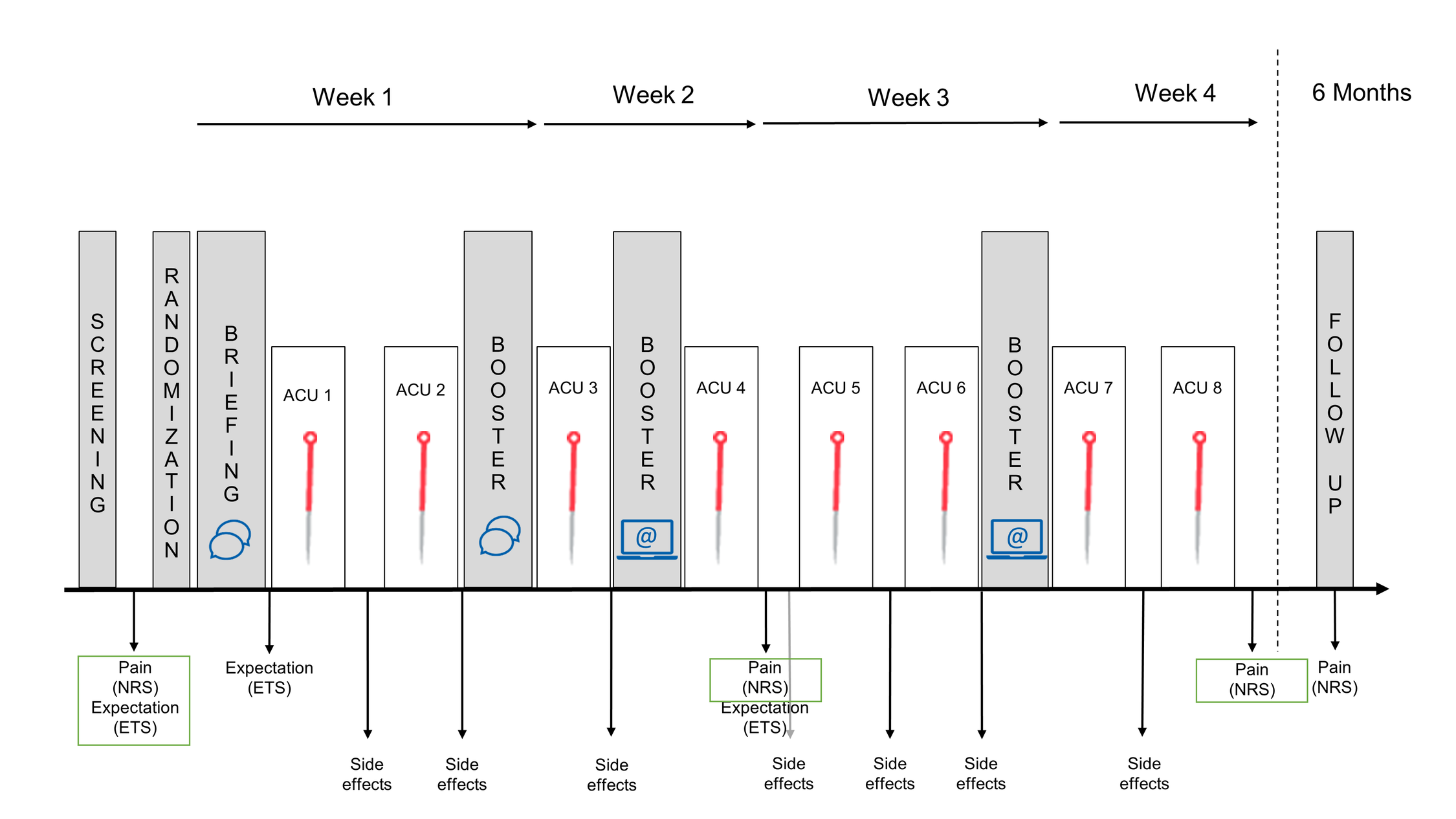

Supplement: S1 Fig — (TIF) [file pone.0268646.s001.tif]
