# Supplementary material for: Do patients’ pre-treatment expectations about acupuncture effectiveness predict treatment outcome in patients with chronic low back pain? A secondary analysis of data from a randomised controlled clinical trial
Source: PLoS One. 2022 May 20;17(5):e0268646. doi: 10.1371/journal.pone.0268646 (PMC9122231; doi:10.1371/journal.pone.0268646)
Supplement: S1 Table — (PDF) [file pone.0268646.s002.pdf]

S1 Table. Pearson correlations to test for the associations of all relevant variables and change in pain intensity as primary outcome.

	Change in pain intensity after treatment			Change in pain intensity after session four		
	r	SE	p-value	r	SE	p-value
**Gender**	-0.145	0.084	0.084	-0.058	0.084	0.489
**Age**	-0.090	0.084	0.281	0.010	0.085	0.931
**Previous experience**						
Previous acupuncture treatment	0.016	0.085	0.853	-0.025	0.085	0.772
Number of sessions of last treatment	0.090	0.130	0.468	0.010	0.130	0.914
Success of last acupuncture treatment	-0.170	0.132	0.215	-0.090	0.133	0.512
**Pain**						
Pain intensity						
before treatment	-0.250	0.082	0.003	-0.400	0.077	< 0.001
after session 4	0.340	0.080	< 0.001	0.620	0.066	< 0.001
after session 8	0.770	0.054	< 0.001	0.260	0.082	0.002
Pain bothersomeness before treatment	-0.140	0.084	0.102	**-0.230**	0.082	**0.007**
Duration of pain	-0.050	0.084	0.593	0.060	0.084	0.486
**ETS Expectation**						
before treatment	-0.150	0.084	0.082	**-0.270**	0.082	**0.002**
after session 4	-0.130	0.084	0.116	**-0.320**	0.080	**< 0.001**
**Self-reported health**						
PROMIS Anxiety	-0.070	0.084	0.385	**-0.150**	0.084	**0.078**
PROMIS Depression	-0.030	0.084	0.756	-0.130	0.084	0.135
PROMIS Ability to participate in social roles	-0.050	0.085	0.565	-0.010	0.085	0.909
PROMIS Fatigue	-0.040	0.084	0.646	-0.090	0.084	0.288
PROMIS Pain Interference	0.050	0.085	0.565	0.040	0.085	0.608
PROMIS Physical function	-0.030	0.085	0.751	-0.080	0.084	0.330
PROMIS Sleep disturbance	0.020	0.085	0.858	-0.050	0.085	0.543
**Personality traits**						
LOT-R Optimism	0.110	0.085	0.193	0.05	0.085	0.562
LOT-R Pessimism	-0.090	0.085	0.286	0.01	0.085	0.870
**Expectation briefing group**	0.130	0.084	0.121	0.03	0.084	0.685
**Side effect briefing group**	0.080	0.084	0.324	0.05	0.084	0.590
