# Supplementary material for: Do patients’ pre-treatment expectations about acupuncture effectiveness predict treatment outcome in patients with chronic low back pain? A secondary analysis of data from a randomised controlled clinical trial
Source: PLoS One. 2022 May 20;17(5):e0268646. doi: 10.1371/journal.pone.0268646 (PMC9122231; doi:10.1371/journal.pone.0268646)
Supplement: S2 Table — (PDF) [file pone.0268646.s003.pdf]

S2 Table. Sensitivity analysis of linear regression for change in pain intensity as primary outcomes and patient expectation as predictor additionally adjusted for expectation and side effect briefing group.

	**Change in pain intensity after treatment session 4**				
	β	SE	p-value	R^2	n
**Expectation at baseline (adjusted)**					
Total	-0.265	0.041	**0.006**	0.070	139

Significant results are marked in bold. Negative sign indicates an impact of expectations on an improvement in pain intensity.

*Analysis was adjusted for pain bothersomeness, anxiety, expectation briefing group and side effect briefing group.
