# Supplementary material for: Do patients’ pre-treatment expectations about acupuncture effectiveness predict treatment outcome in patients with chronic low back pain? A secondary analysis of data from a randomised controlled clinical trial
Source: PLoS One. 2022 May 20;17(5):e0268646. doi: 10.1371/journal.pone.0268646 (PMC9122231; doi:10.1371/journal.pone.0268646)
Supplement: S3 Table — Significant results are marked in bold. Negative sign indicates an impact of expectations on an improvement in pain intensity. *Analysis was adjusted for pain bothersomeness, anxiety, expectation briefing group, side effect briefing group, PROMIS, LOT, PSM and side effect score. (PDF) [file pone.0268646.s004.pdf]

S3 Table. Sensitivity analysis of linear regression for change in pain intensity as primary outcomes and patient expectation as predictor additionally adjusted for expectation and side effect briefing group, PROMIS, LOT, PSM and side effects.

	**Change in pain intensity after treatment session 4**				
	β	SE	p-value	R^2	n
**Expectation at baseline (adjusted)**					
Total	-0.257	0.039	**0.005**	0.184	132

Significant results are marked in bold. Negative sign indicates an impact of expectations on an improvement in pain intensity.

*Analysis was adjusted for pain bothersomeness, anxiety, expectation briefing group, side effect briefing group, PROMIS, LOT, PSM and side effect score.
